# Inferring transient dynamics of human populations from matrix non-normality

**DOI:** 10.1007/s10144-018-0620-y

**Published:** 2018-06-05

**Authors:** Alex Nicol-Harper, Claire Dooley, David Packman, Markus Mueller, Jakub Bijak, David Hodgson, Stuart Townley, Thomas Ezard

**Affiliations:** 1 Ocean and Earth Science, National Oceanography Centre, University of Southampton Waterfront Campus, Southampton, UK; 2 Environment and Sustainability Institute, College of Engineering, Mathematics and Physical Sciences, University of Exeter in Cornwall, Penryn Campus, Cornwall, UK; 3 Department of Social Statistics and Demography, ESRC Centre for Population Change, Southampton Statistical Sciences Research Institute, University of Southampton, Southampton, UK; 4 Centre for Ecology and Conservation, College of Life and Environmental Sciences, University of Exeter in Cornwall, Penryn Campus, Cornwall, UK

**Keywords:** Damping ratio, Europe, Eurostat, Human demography, Population projection matrix, Pseudospectra

## Abstract

In our increasingly unstable and unpredictable world, population dynamics rarely settle uniformly to long-term behaviour. However, projecting period-by-period through the preceding fluctuations is more data-intensive and analytically involved than evaluating at equilibrium. To efficiently model populations and best inform policy, we require pragmatic suggestions as to when it is necessary to incorporate short-term transient dynamics and their effect on eventual projected population size. To estimate this need for matrix population modelling, we adopt a linear algebraic quantity known as non-normality. Matrix non-normality is distinct from normality in the Gaussian sense, and indicates the amplificatory potential of the population projection matrix given a particular population vector. In this paper, we compare and contrast three well-regarded metrics of non-normality, which were calculated for over 1000 age-structured human population projection matrices from 42 European countries in the period 1960 to 2014. Non-normality increased over time, mirroring the indices of transient dynamics that peaked around the millennium. By standardising the matrices to focus on transient dynamics and not changes in the asymptotic growth rate, we show that the damping ratio is an uninformative predictor of whether a population is prone to transient booms or busts in its size. These analyses suggest that population ecology approaches to inferring transient dynamics have too often relied on suboptimal analytical tools focussed on an initial population vector rather than the capacity of the life cycle to amplify or dampen transient fluctuations. Finally, we introduce the engineering technique of pseudospectra analysis to population ecology, which, like matrix non-normality, provides a more complete description of the transient fluctuations than the damping ratio. Pseudospectra analysis could further support non-normality assessment to enable a greater understanding of when we might expect transient phases to impact eventual population dynamics.

## Introduction

Our world is in constant flux, so populations are never at equilibrium. Population dynamics are altered by ongoing and abrupt processes, both immediately and over longer timescales, diverting trajectories from the paths they would otherwise follow. Transient fluctuations such as baby booms dampen away, leaving population size modified by a process known as momentum (Keyfitz 1971; Espenshade and Tannen 2015)—or more generally and formally, inertia. Inertia occurs when unstable population structures cause eventual population size to be larger or smaller than if projected from a stable initial stage structure; momentum is the special case for stationary populations with zero growth (Koons et al. 2007). Given the importance of population projections to national and global development policies (UN 2015), we need a better understanding of how transients affect population dynamics in the short- and long-term (Osotimehin 2011), and how responses are shaped by environmental and social factors at a range of spatial scales (Hastings 2004; Harper 2013).

Although equilibrium approximations are useful in the absence of complete population knowledge at each point in time (Caswell 2000), there is increasing recognition that systems are dynamic entities for which short-term transient effects must also be considered as fundamental aspects of ecological dynamics (Hastings 2004; Ezard et al. 2010; Stott et al. 2010), explaining approximately half of the variation in growth rates in comparative studies of plants (Ellis and Crone 2013; McDonald et al. 2016). This is especially important when shorter timescales are of greater applied relevance (Hastings 2004; Ezard et al. 2010), or when repeated disturbances prevent populations from settling to equilibrium behaviour (Townley and Hodgson 2008; Tremblay et al. 2015). In human populations, gradual demographic transitions (from high to low rates of mortality and fertility) are a major driver of transient phenomena (Blue and Espenshade 2011), over and above abrupt disturbances such as wars and pandemics. In deterministic models—as used here for conceptual clarity (see Ezard et al. 2010)—transients can be considered deterministic responses to stochastic events (Stott et al. 2010). This allows setting of bounds, which “help to create an envelope of possible future population scenarios around the mean, long-term prediction” (Townley and Hodgson 2008, p. 1836), aiding in the incorporation of at least some aspects of uncertainty into near-term estimates for a given population structure.

We know that transients occur when disturbances destabilise population structure, causing deviation from the proportional composition that balances different groups’ varying contributions to population growth or decline (Townley and Hodgson 2008). Precise predictions of transient dynamics require detailed and frequent updating of population structures, which is typically data-intensive, as it requires making specific, fine-grained assumptions about the future (Townley et al. 2007). In long-lived organisms with age-dependent schedules of maturation and reproduction, such as modern humans *Homo sapiens*, structuring is by age: stable age structure is determined by the age-structured life table (Caswell 2001). Given that transient analysis “produce[s] output which is complicated, and difficult to define succinctly” (Yearsley 2004, p. 245), it would be useful to have diagnostic tools to indicate if it is desirable to perform further analyses on transients.

Asymptotic and transient behaviour can be disentangled in matrix population modelling (Caswell 2001). Population projection matrices (PPMs) are built using (st)age-specific rates of reproduction and transition between life cycle stages (vital rates), to project population structures over time. The ‘eigendecomposition’ of a matrix determines the spectrum (set of eigenvalues) and ‘natural directions’ (set of eigenvectors) of a matrix,^1^ and is used to analyse the model: for PPMs, the dominant eigenvalue gives the asymptotic growth rate, and its associated right and left eigenvectors determine the stable (st)age structure and (st)age-specific reproductive values, respectively. Subdominant eigendata pertain to transient responses, with decreasing influence over time following disturbance from the stable (st)age structure (Caswell 2001).

**Fig. 1:**
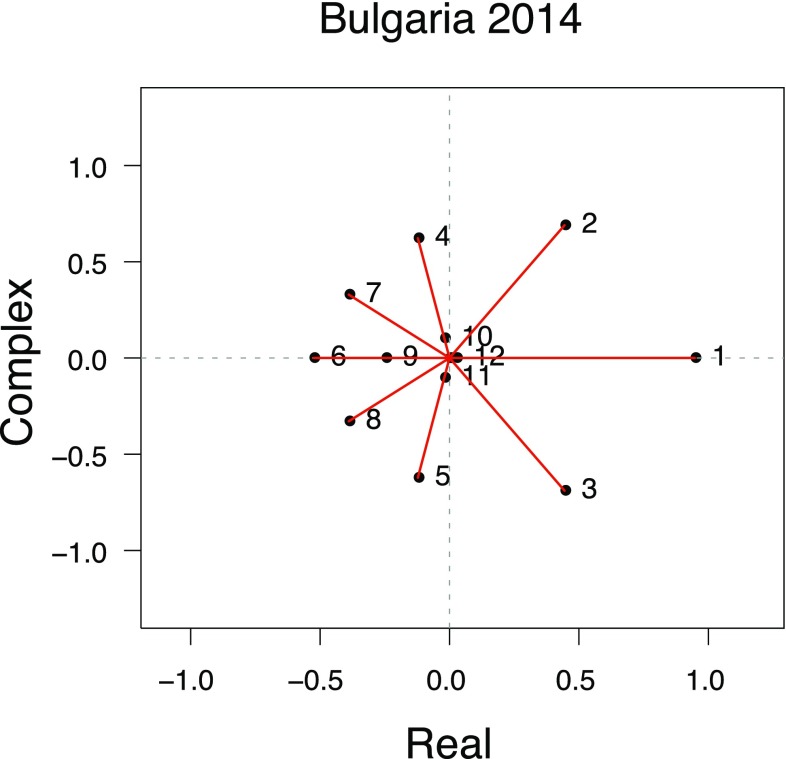
Eigenvalue spectrum for Bulgaria in 2014. Numbers correspond to eigenvalues ordered by magnitude, which is calculated as the length of the vector joining each point to the origin (shown in red). Eigenvalues 13–18 lie on the origin. Note the similarity in magnitude of, say, the 4th eigenvalue to that of the 2nd

The classical metric of the duration of this decreasing influence is the damping ratio, which is calculated as the ratio of the dominant eigenvalue divided by the absolute value of the subdominant eigenvalue (Caswell 2001). As a measure of ‘intrinsic population resilience’ to transient deviations (with a higher value suggesting a shorter recovery time), the damping ratio has been shown to be useful in comparative demography (Stott et al. 2011). However, it is methodologically limited, because rather than bounding the duration of transient dynamics, it actually measures the asymptotic rate at which transients decay. As such, it correlates weakly with convergence times of realistic population projections (Stott et al. 2011) because transient dynamics are not determined solely by the largest two eigenvalues, as the damping ratio assumes, but rather by the whole set. Figure 1 shows an eigenvalue spectrum for a PPM for Bulgaria in 2014, demonstrating that many of the lower eigenvalues can have magnitudes similar to the subdominant one—highlighting how much information for predicting transient dynamics is lost when focusing solely on the damping ratio. More integrative measures of eigenvalue variation have the potential to increase the accuracy of transient dynamic predictions (cf. Crone et al. 2013).

In population ecology, transients are the result of an initial population vector being propagated through a population projection matrix. The focus of efforts into transient fluctuations has most often centred on how the population structure at a given point in time differs from the stable age distribution [reviewed by Williams et al. (2011)]. As individuals at different developmental (st)ages have different mortality and fertility rates, the discrepancy between observed and stable population structures causes the aggregated population growth rate to change despite constant demographic rates (Koons et al. 2005; Ezard et al. 2010; Stott et al. 2011). This focus on population structures represents a single side of the same coin—a given initial condition can have very different transient dynamics depending on the matrix through which it is projected. This leads to asking whether there are properties of the PPM that can indicate a system’s propensity to exhibit amplificatory dynamics.

It has long been recognised within mathematics that transient dynamics depend on a matrix characteristic known as ‘normality’ (Elsner and Paardekooper 1987; Trefethen and Embree 2005). If a matrix is normal its properties are fully determined by eigendata (Trefethen and Embree 2005), the set of basis values and vectors that describe the core properties of the system. While undoubtedly valuable (Caswell 2001; Hodgson et al. 2006; Crone et al. 2011), eigendata are an asymptotic description and therefore cannot capture all dynamical aspects of populations’ short- and medium-term trajectories as determined by asymmetric, non-normal PPMs. Transient effects are limited in normal systems, but can be substantial (Trefethen 1997) and potentially dominating (Townley et al. 2007) in non-normal ones. A key challenge then is to find and understand simple measures of non-normality that might predict and explain links between matrix asymmetry and transient dynamics in population ecology and evolutionary demography.

Here, we apply non-normality metrics to PPMs. Human populations are particularly susceptible to transients as a result of culture and geopolitics inducing strong cohort effects (Ezard et al. 2010), in addition to long lifespan (Koons et al. 2005, 2007). Momentum will dominate long-term population dynamics in Africa and Asia due to high uncertainty and variability in fertility and mortality rates (Azose et al. 2016), and can be expected to account for over half of all population growth in developing countries from 1995 to 2100 (Bongaarts 1994). We used Eurostat data for 1960 to 2014 to build over 1000 PPMs of country–year combinations. After showing that non-normality has generally increased in these PPMs over time, we use multivariate analyses to highlight the dependencies among the facets of matrix non-normality and classical ecological population dynamic metrics. Our three non-normality metrics correlate well with transient indices, but not with the damping ratio. These patterns are best drawn out through an important distinction between non-normality for the system as a whole, combining asymptotic and transient dynamics, and that for the scaled system, when asymptotic growth rate is factored out. Finally, we also introduce to population ecology the technique of pseudospectra analysis (Trefethen and Embree 2005), originally derived from applications in fluid dynamics (Trefethen et al. 1993), which should prove helpful in the incorporation of non-normality assessment into matrix population modelling.

## Methods

### Data

We used the Eurostat database (http://ec.europa.eu/eurostat) to collect secondary data on age-specific female population sizes, births and deaths, for the 45 European countries with complete population data for any subset of years 1960–2014 (range 3–55 years, 6 complete sets, mean 28 years). The variables are provided in single-year age classes, up to the oldest age recorded or an arbitrary ‘*x* years and over’ category. Following standard human demography protocols (e.g., Keyfitz and Flieger 1968, 1971, 1990; Wiśniowski et al. 2016), we aggregated into 18 5-year bins, up to ‘85 years and over’. Total births are available separated by babies’ sex from 2007 only, so we estimated female births by taking the ‘sex ratio at birth’ values for the relevant countries and years from the World Bank Databank (http://databank.worldbank.org/data/reports.aspx?source=gender-statistics), and calculating their grand mean. We removed 121 country–year combinations that had five or more consecutive zero deaths across single-year classes—including all data for Andorra, Liechtenstein, and San Marino—since this is either suggestive of inaccurate data collection and/or curation, or related to impractical small population counts. This left 1,120 country–year combinations from 42 countries for matrix construction. Note that all available years were used, so PPMs could overlap in their timeframes; for example, where data were available for both 2001 and 2002, there would be a matrix using 2001 data projecting to 2006, and another from 2002 to 2007.

### Matrices

For each available country–year combination, we projected the observed population at year 0 to year 5, by premultiplying the initial population vector, **n**_0_, by its corresponding PPM, **A**: i.e., **n**_t+5_ = **An**_t._ The timestep is 5 years due to the data being aggregated into 5-year bins; an individual which is 0–4 years old at year 0 will be 5–9 years old after projection. The initial population vectors had 18 entries representing the observed population structure across the 5-year age bins; the PPMs were of dimension 18 × 18. Each matrix was generated via the following approximations for each bin:

- $$survival~i.e.,~progression=1 - \left( {\frac{{5~ \times ~deaths}}{{population~size}}} \right)$$
  - included along the matrix subdiagonal, for bins 0–4 to 80–84
- $$85+survival~i.e.,~stasis=1 - \left( {\frac{{5~ \times ~deaths}}{{population~size}}} \right)$$
  - included in the final entry of the matrix diagonal
- $fertility=\left( {\frac{{5~ \times ~births}}{{population~size}}} \right)\left( {\sqrt {survival ( {maternal} )} } \right)\left( {\sqrt {survival\left( {0 - 4} \right)} } \right)$ (following the birth-flow approximation of Morris and Doak 2002)
  - included along the top row of the matrix.

Note that negative survival values, which arose when quintupled deaths exceeded population size, were replaced with zero. Additionally, survival was calculated separately for infants under 1 year and children aged 1–4 years—since deaths are much higher in the former stage—and then combined as follows:

$$survival(0 - 4)=(0.2 \times survival\;(0))+(0.8 \times survival\;(1 - 4)).$$

For each matrix we computed:

- eigenvalues, *λ*_*n*_, using base R’s eigen() function,
- $$damping~ratio=~{\raise0.7ex\hbox{${{\lambda _1}}$} \!\mathord{\left/ {\vphantom {{{\lambda _1}} {\left| {{\lambda _2}} \right|}}}\right.\kern-0pt}\!\lower0.7ex\hbox{${\left| {{\lambda _2}} \right|}$}}$$ (Caswell 2001),
- case-specific reactivity, the relative population size after one projection interval, standardised for *λ*_1_, $=\|{\mathbf{\hat {A}}}{{\mathbf{n}}_0}\|_{1}$ (Stott et al. 2011) where ${\left\| {} \right\|_{1}}$is the one-norm (the sum of the modulus of the entries) of a vector, ${\mathbf{\hat {A}}}=~{\mathbf{A}}/{\varvec{\lambda}_1}$, and **n**_0_ is the initial population structure scaled such that it sums to 1 (giving the proportions of the population in each 5-year age bin),
- inertia, the relative population size after the transient period (here defined as 100 timesteps i.e., 500 years) $=\|{{\mathbf{n}}_{100}}\|_{1}$ where ${\left\| {} \right\|_1}$ is the one-norm (sum) of a vector and ${{\mathbf{n}}_{100}}={{\mathbf{\hat {A}}}^{100}}{{\mathbf{n}}_0}~$,
- various non-normality metrics, discussed below.

Note that Stott et al. (2011) differentially name positive and negative transient indices, such that a negative value of our ‘case-specific reactivity’ would correspond to ‘case-specific first timestep attenuation’ in their treatment.

### Non-normality

Elsner and Paardekooper (1987) reviewed matrix non-normality and presented four main metrics, one intuitive definition (distance from the set of normal matrices) and three pragmatic implementable suggestions (Table 1). All three metrics have their foundations in AA* rather than just **A**, and tackle the discrepancy between AA* and A*A to reveal the asymmetry of **A**. The Henrici metric uses the Frobenius norm of A*A, while the Frobenius and Ruhe metrics use the eigendata of A*A, also known as the singular value decomposition of **A**, as previously introduced to evolutionary biology (Townley and Ezard 2013). The singular value decomposition of **A** is the eigendecomposition of AA*, yielding an alternative set of basis values and vectors. If **A** is symmetric and normal, the singular value and eigen-decompositions are the same. With increasing asymmetry of the PPM, the singular value and eigen-decompositions diverge.

**Table 1:** Non-normality metrics

Non-normality metric	Formula	Code in R	Explanation
Frobenius	$\sqrt {{\|{{\mathbf{A}}^{\text{*}}}{\mathbf{A}} - {\mathbf{A}}{{\mathbf{A}}^{\text{*}}}\|}_{F}}$	> sqrt(norm((Conj(t(A))%*%A) − (A%*%Conj(t(A))), type=‘F’)) Or > sqrt(norm((Conj(t(Re(Â)))%*%Re(Â)) − (Re(Â)%*%Conj(t(Re(Â)))), type=“F”))	One of the main conditions defining matrix normality is the equality A^*^A = AA^*^; this metric provides a measure of non-normality by quantifying the discrepancy between **A** and A^*^
Henrici	$$\sqrt {\|{{\mathbf{A}}\|_F}^{2} - {{\varvec{\Sigma}}}_{{k=1}}^{n}{{\left| \lambda \right|}^2}}$$	> Re(sqrt(norm(A, type=“F”)^2 − sum(abs(eigen(A)$values)^2))) Or > Re(sqrt(norm(Re(Â), type=“F”)^2 − sum(abs(eigen(Re(Â))$values)^2)))	This metric considers all eigenvalues of matrix **A**, and is in fact a rearrangement of the Frobenius norm of A^*^A. It quantifies non-normality since “**A** is normal if and only if [formula] = 0” (Henrici 1962, p. 27)
Ruhe	$$ma{x_k}\left| {{\sigma _k} - \left| {{\lambda _k}} \right|} \right|$$	> max(svd(A)$d − abs(eigen(A)$values)) Or > max(svd(Re(Â))$d − abs(eigen(Re(Â))$values))	Maximum difference between singular value and associated absolute eigenvalue: close to normal if similar, increasingly non-normal with distance. The singular value decomposition is the eigendecomposition of AA^*^, yielding an alternative set of basis values and vectors

**A** is a matrix (${\mathbf{\hat {A}}}$ if standardised); A^*^ is the conjugate transpose of **A**; $\|$ is a scalar magnitude; $\|$ is a matrix norm—subscript F specifies the Frobenius norm: $\surd$(Σ_*j*_Σ_*i*_|*a*_*ij*_|^2^) (*a*_*ij*_ is a matrix entry, where *i* denotes row and *j* denotes column); *λ*_*k*_ is the *k*th eigenvalue (ordered by decreasing magnitude) of total *n* (*n* = matrix dimension); *σ*_*k*_ is the *k*th singular value (ordered corresponding to eigenvalues) (Elsner and Paardekooper 1987; Henrici 1962; Ruhe 1975)

In order to isolate transient effects from the overall system, we present the results obtained by using standardised (${\mathbf{\hat {A}}}$) matrices in addition to raw ones (**A**); scaling by *λ*_1_ removes differences in dynamics that result from populations increasing or decreasing (Koons et al. 2005; Townley and Hodgson 2008; Stott et al. 2011). While Elsner and Paardekooper (1987) additionally present alternative versions of the Frobenius and Henrici metrics using the spectral rather than Frobenius norm, we chose to limit our analyses to the Frobenius norm only, since it simplifies the interpretation of the Henrici metric (see Trefethen and Embree 2005, pp. 444–445; Table 1). To visualise non-normality over time, we generated generalised additive mixed models (GAMMs) with year as a smoothed fixed effect, controlling for country as a random effect. We used the ‘gamm4’ package (Wood and Scheipl 2016), fitted with family ‘Gamma’ and the ‘identity’ link function.

### Multivariate analyses

Linear correlations were calculated using Spearman’s rank correlations; those presented were significant with *P* < 0.05 and are given to 2 decimal places. Principal component analysis was used to assess relationships among metrics. This was conducted (using base R’s prcomp() function) for both raw and standardised matrices, with scaled and centred non-normality metrics and a range of relevant variables (see Table 2 for justifications and definitions). We generated biplots from the informative principal components—defined as those with eigenvalues exceeding 1, after ‘conservative’ bias correction using the 95th percentile in parallel analysis (Peres-Neto et al. 2005) using the ‘paran’ package (Dinno 2012). We list loadings that exceeded 10% of each axis, in the order of decreasing importance.

**Table 2:** Variables used in the principal component analysis

Variable	Justification	Definition
Year	Transient dynamics were expected to change over time	N/A
Asymptotic growth rate, *λ*_1_	A component of total population growth rate A key matrix output The numerator of the damping ratio	The rate at which the population would grow or decline in the absence of transient dynamics The dominant eigenvalue of a PPM
Damping ratio	A metric originally formulated to measure the duration of transient impact	The dominant eigenvalue divided by the absolute value of the subdominant eigenvalue (which can be a complex number)—see “Matrices”
Reactivity	An index of short-term transient impact	Relative population size, after scaling out the asymptotic growth rate, in the first timestep—see “Matrices”
Inertia	An index of long-term transient impact	Relative population size, after scaling out the asymptotic growth rate, after 100 timesteps—see “Matrices”
Frobenius non-normality	Metric under consideration	See Table 1
Henrici non-normality	Metric under consideration	See Table 1
Ruhe non-normality	Metric under consideration	See Table 1

The statistical software ‘R’ (version 3.3.2, R Development Core Team 2016) was used for all analyses and figures, along with the ‘R ColorBrewer’ package (Neuwirth 2014) for the latter.

## Results

**Fig. 2:**
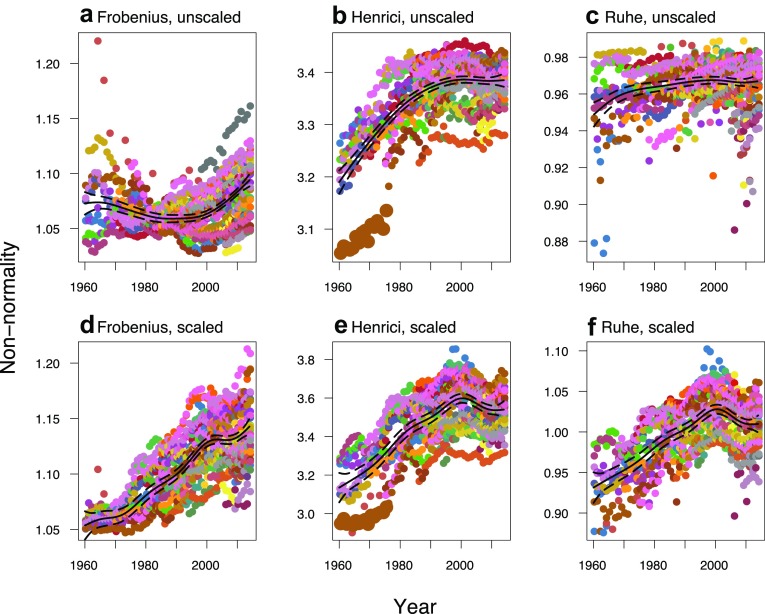
Non-normality over time, measured by three different metrics—from left to right: Frobenius, Henrici, Ruhe. Top row shows raw matrices, and bottom row standardised ones. Points are coloured by country. Solid lines are GAMM estimates, with dashed lines showing their 95% confidence intervals

Figure 2 shows how non-normality in European human populations has increased over time. The top row illustrates non-normality of the whole system: raw matrices describe both asymptotic and transient dynamics. In that context: the Frobenius metric changed little over the time period; the Henrici metric increased up to a plateau beginning around 1990 (with low outliers including Portugal 1960–1975, enlarged on the figure and examined below); the Ruhe metric showed an almost flat relationship.

**Fig. 3:**
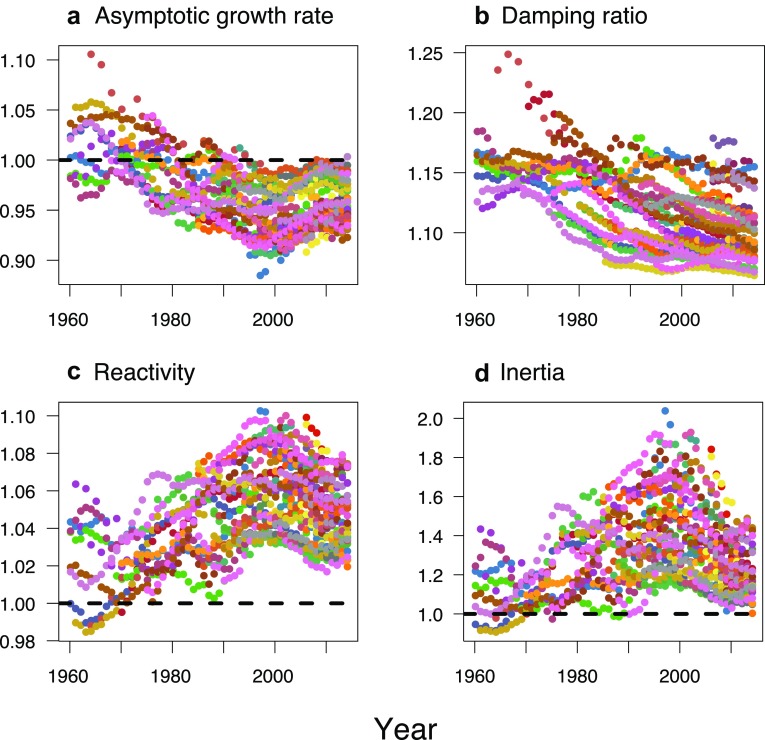
Ecological measures of population dynamics over time. Points are coloured by country. Asymptotic growth rate is the dominant eigenvalue, with the dashed line showing *λ*_1_ = 1 i.e., no population change—above the line is population growth; below, decline. For definitions of the other measures, refer to “Methods”. For reactivity and inertia, the dashed lines divide the plot into transient growth (> 1) and decline (< 1)

Scaling focuses on transient effects by factoring out the effect of *λ*_1_. This increased the mean value of all non-normality metrics, by almost 4% each. Comparing the bottom and top rows of Fig. 2 shows that the shape of the estimated GAMM curves also changed, in terms of intercept, slope, and variance patterns. This can be coarsely explained by a systematic change in *λ*_1_: the annual mean dropped below 1 in 1975 and remained so for the rest of the time period (see Fig. 3a). The Frobenius metric (Fig. 2a, d) shows how higher *λ*_1_ values before 1975 were pushing the curve up, while lower values afterwards pulled it down. The overall effect resulted in similar increases over time across the scaled non-normality metrics (which were pairwise correlated with one another at *ρ* > 0.78). However, in contrast to the smooth increase in the scaled Frobenius metric, the Henrici and Ruhe metrics both show a peak around 2000—close to that of the transient indices (see Fig. 3c, d)—and appear to plateau by the end of the time series.

Even where scaling did not change the overall pattern, as with the Henrici metric, increased variance allows an improved visualisation of dynamics. Additionally, outliers tended to become less distinct, although corresponding country–years are still distinguishable as bounds on variation in top and bottom rows of Fig. 2. The outlying line of Portugal 1960–1975 on the plots of the Henrici metric (enlarged points in Fig. 2b, e) corresponds to matrices with very low old-age survivals and zero 85 + stasis.

Figure 3 shows that both *λ*_1_ and the damping ratio decreased over time. The transient indices of reactivity and inertia were strongly correlated with one another (*ρ* = 0.93), both peaking around 1995. Furthermore, values for both exceeded 1 for over 97% of matrices, revealing a propensity for amplifying transient growth rather than decline, with the latter being restricted to prior to 1971 for reactivity and 1991 for inertia. The transient indices were positively correlated with all scaled non-normality metrics (*ρ* > 0.52).

**Fig. 4:**
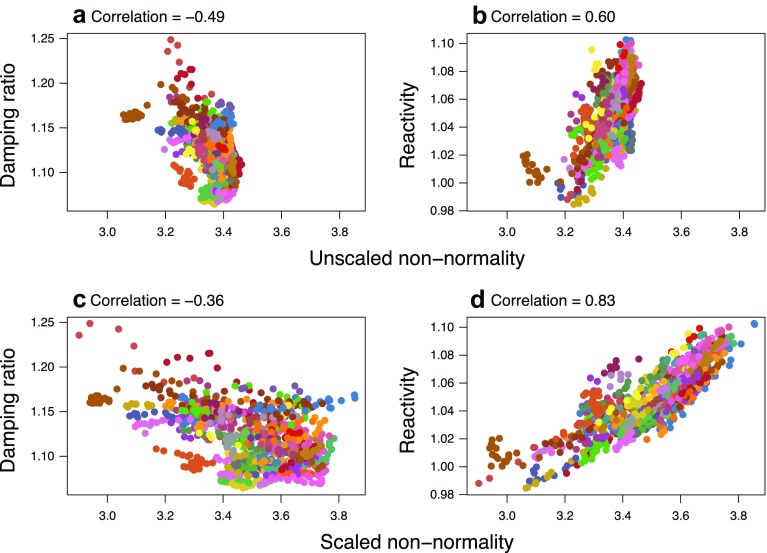
The Henrici non-normality metric, unscaled (top) and scaled (bottom), against damping ratio (left) and reactivity (right). Points are coloured by country. Simple Spearman’s rank correlation coefficients are given above each plot as a visualisation aid

Of the three non-normality metrics, Henrici changed the least with matrix standardisation; the scaled and unscaled versions were correlated at *ρ* = 0.82. Nevertheless, Fig. 4 shows that scaling still altered the Henrici metric’s relationships with ecological measures of population dynamics. It decreased the strength of the relationship between non-normality and damping ratio (Fig. 4a, c), such that there was only a slight correlation with the effect of *λ*_1_ removed; this is unsurprising given *λ*_1_ is the numerator of the damping ratio. In contrast, scaling increased the strength of the relationship between non-normality and reactivity (Fig. 4b, d), such that high values of scaled non-normality were a good predictor of strong immediate transient growth.

**Fig. 5:**
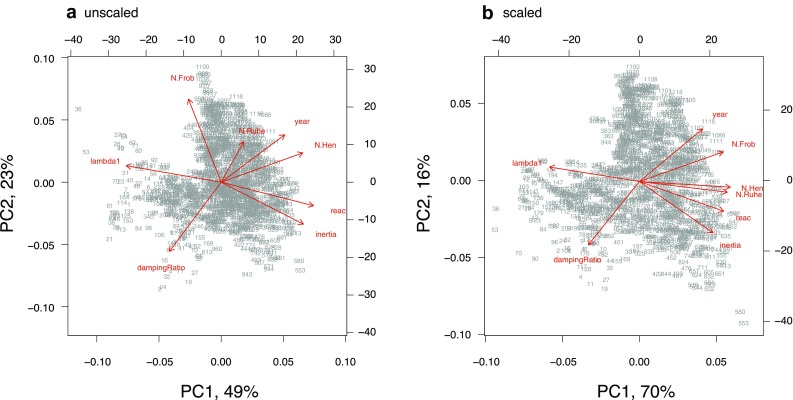
Biplots of principal component analysis on non-normality metrics (prefix ‘N’) and ecological measures of population dynamics. **a** Using unscaled metrics; **b** scaled. *Frob* Frobenius, *Hen* Henrici, *lambda1 λ*_1_ i.e., dominant eigenvalue, *reac* reactivity

Principal component analysis allowed more in-depth investigation of interrelationships among the variables, visually represented as biplots in Fig. 5. Using the unscaled non-normality metrics, the two significant principal components explained 72% of the variance. The first principal component loaded onto *λ*_1_ (negatively), and transient indices, the Henrici metric, and year (positively). The second loaded onto the Frobenius metric (positively), damping ratio (negatively), and year again (positively). Note that the Ruhe metric is not represented by either of the significant principal components. Using the scaled non-normality metrics, the two significant principal components explained more of the variance (86%) than the unscaled case. Loadings differed, but directions did not: the first principal component loaded onto the Henrici metric, *λ*_1_, the Ruhe metric positively, the Frobenius metric, and transient indices; the second component loaded onto damping ratio, year, and inertia again, but this time negatively.

Scaling moved all non-normality metrics into the same part of the plot (in Fig. 5b), whereas when unscaled, the Frobenius and Henrici metrics were almost orthogonal to each other (in Fig. 5a). The Frobenius and Ruhe metrics appeared to be most susceptible to asymptotic growth rate, moving more than the other variables when the effect of *λ*_1_ was removed; this reiterates the relatively low sensitivity of the Henrici metric to scaling. In both plots the damping ratio was orthogonal to the axis with *λ*_1_ and transient indices (unscaled plot) or non-normality (scaled), suggesting that it describes something fundamentally different to *both* asymptotic *and* transient dynamics—which should perhaps be unsurprising since it is supposedly a measure of duration rather than amplitude. Two groups of points (labelled as: 36, 53, 70; and 553, 580) are notable outliers on both biplots: the former represent Iceland in the 1960s; the latter Bulgaria in the late 1990s.

## Discussion

This is, to the best of our knowledge, the first comprehensive continental-scale comparative assessment of the susceptibility of human populations to transient dynamics. We quantified this transient potential using non-normality metrics: overall, these increased for European populations between 1960 and 2014 (Fig. 2). The patterns of non-normality metrics were correlated with transient indices (Figs. 2, 3): relationships were strong and positive, with the peaks in the scaled Henrici and Ruhe metrics echoed in those for reactivity and inertia—implying increasing influence of transient dynamics on these populations. Although we caution against the potential loss of information in restricting analyses to a single measure of non-normality, where a streamlined evaluation is desired we particularly recommend the Henrici metric, since in our study it proved to be least affected by the scaling issue and most strongly correlated with transient indices.

Focusing on these transient indices, we found a very strong and significant correlation between reactivity (transient change in population size after one timestep) and inertia (asymptotic change in population size due to transience), as did Stott et al. (2011). Our transient indices rarely yielded attenuation, i.e., values smaller than one, which reflect decreases relative to the asymptotic trajectory. In contrast, using the same metrics on orchids, Tremblay et al. (2015) showed transient decline to be much more common than amplification; this suggests that the western human populations that are most common in our database tend towards transient increases, while plants may more often decrease. While we found a greater likelihood of transient increase when populations were declining overall (and vice versa), since both transient indices were opposed to *λ*_1_ (Fig. 5), the opposite was found in a study of over 100 plant species, where faster-growing populations tended towards greater reactivity (along with other measures of transience; Stott et al. 2010). Stott et al. (2010) argued from their results that vital rates impacted short- and long-term dynamics similarly, but pointed out that animal populations including humans appear to be more sensitive to initial conditions.

The opposition of short- and long-term dynamics is further drawn out in the contrast of decreasing *λ*_1_ through time, whereas reactivity and inertia peak around the millennium. The first observation is increasingly recognised: for many countries worldwide, and especially in Europe, a ‘second demographic transition’ is underway, with total fertility rate dropping below replacement, driving population decline in the absence of immigration (Harper 2013; van Daalen and Caswell 2015). Any reason for a peak in transience is less obvious. Lutz et al. (2003) found that “for the [then] 15 member countries of the EU, low fertility brought the population to the turning point from positive to negative momentum around the year 2000” (p. 1991). However, inspection of country-stratified data suggests that the humps are a combination of different types of trajectory, rather than all countries peaking simultaneously. Perhaps some are related to preceding and ongoing disturbances such as the dismantling of the socialist economic model in Central and Eastern Europe (Sobotka 2002), the reunification of Germany (1989), and the armed conflict in the former Yugoslavia (1991–1999). As a specific example, Bulgaria’s economic instability during the 1990s could have driven the transient effects suggested by the PPMs for 1997 and 1998, which were outliers on the biplots and had the highest values for the scaled Henrici metric.

Returning to the non-normality metrics, we found all three measures to have similar temporal trends once the effect of declining asymptotic growth had been factored out. This follows Stott et al.’s (2011) recommendation that transient analyses are more usefully performed on standardised matrices. When studying the whole system, using raw matrices, the different non-normality metrics told varying stories: Frobenius suggests a negative quadratic relationship, Henrici increases to a plateau, and Ruhe shows very little change. This impact of *λ*_1_ is especially notable given the relatively small range of values seen across human populations as opposed to other animals or plants: this study saw 0.89–1.11, compared to 0.80–1.12 within one metapopulation of marmots (Ozgul et al. 2009), and approximately 0.7–2.1 across 20 plant species (Crone et al. 2013). The effect of *λ*_1_ should therefore be acknowledged in all comparative studies of transients (Stott et al. 2011).

Furthermore, we suggest that longitudinal (as well as comparative) studies should consider the potential for varying non-normality across datasets. Both overall trends and turning points illustrate that non-normality cannot be considered static for a given country, rather as changing temporally—perhaps similarly to momentum which is a process that plays out over time (Blue and Espenshade 2011). While a non-normality value for a single matrix reveals little about the impact of the transient at that snapshot in time, its relation to others in the dataset integrate multiple sources and forms of stochasticity with respect to the impact of varying transient dynamics on population trajectories. Historically, the damping ratio has been used to quantify transient impact, but it exhibits orthogonal behaviour to inertia and reactivity (Fig. 5). Over and above the methodological limitations of the damping ratio already discussed, a key consideration is the fact that the damping ratio is a proxy for the duration of transient fluctuations, while reactivity and inertia provide immediate and eventual measures of the transient amplification in population size. It remains to be seen how the three non-normality metrics perform across other systems and stage structures, and whether their interrelationships with population dynamic indices remain consistent. Comparative studies using the COMPADRE and COMADRE demographic databases (Salguero-Gómez et al. 2015, 2016) could prove particularly insightful here.

### Caveats

Matrix outputs are affected by matrix dimension (Tenhumberg et al. 2009), with potential implications for non-normality. A study on cacti found larger matrices to generate lower asymptotic growth rates (Rojas-Sandoval and Meléndez-Ackerman 2013). With our data, single-year matrices (of dimension 85 × 85) generated *λ*_1_ values up to 9% larger or smaller than those from the 18 × 18 matrices used here, with a mean difference of + 3% (unpublished data); we employed the smaller matrices in this study for consistency with standard approaches in human demography and because they capture the vast majority of variation whilst enabling expansion to other regions and time periods for which annual data are not available. Influence of matrix dimension on transients is more contested: while a study of six bird and mammal species with varied life histories found no effect (Koons et al. 2005), a piece of research on pea aphids and another on a wide range of plants found positive correlations (Tenhumberg et al. 2009; Williams et al. 2011). Furthermore, the potential for transients has been found to affect the magnitude of changes in *λ*_1_ with matrix dimensionality (Ramula and Lehtilä 2005). Although Stott et al. (2010) are concerned that such effects could “perhaps [be] signifying a potentially worrying artefact of basic model parameterisation” (p. 302), Ellis (2013) reassures that these relationships are likely to be weaker when considering case-specific transient indices (‘realistic’ scenarios, as here), compared to bounds (extreme hypothetical cases; see Stott et al. 2011).

A further fundamental caveat is the lack of migration among populations, which is increasingly considered essential when modelling human populations (Azose et al. 2016; Willekens 2016). Ozgul et al. (2009) shows how transients unfold differently when incorporating migration between patches in metapopulations. Inclusion of such complexity reveals highly variable transient responses (Espenshade and Tannen 2015, and the unpublished EU study therein), with eminent policy implications.

A more significant limitation to our study is the observation that differing behaviours of non-normality metrics with respect to matrix standardisation remind us that these measures may be well-defined mathematically but less so with relevance to demography. Even in their original formulations, “scalar measures of nonnormality suffer from a basic limitation: Non-normality is too complex to be summarised in a single number” (Trefethen and Embree 2005, p. 446). There is therefore still a need to develop more reliable measures. One response (Gheorghiu 2003) to Elsner and Paardekooper’s (1987) review of non-normality metrics considered scalar instruments to be just one of two ‘major concepts’ in their measurement—the other being pseudospectra analysis.

### A future direction: pseudospectra analysis for population ecology

Pseudospectra are visual representations of non-normality developed by Trefethen and colleagues (Trefethen 1992; Trefethen et al. 1993; Trefethen and Embree 2005) for applications in fluid dynamics, but with the recognition that the techniques also apply to related problems across the mathematical sciences. Trefethen (1997) believes that visual representations aid interpretation by “supplementing the abstract notion of a matrix [with] a picture in the complex plane” (p. 383). He suggested that pseudospectra give matrices ‘personality’, and that they may allow us “to notice things that went unnoticed before” (p. 404). Pseudospectra can now be interrogated via perturbation analysis and transient bound calculation (Townley et al. 2007).

**Fig. 6:**
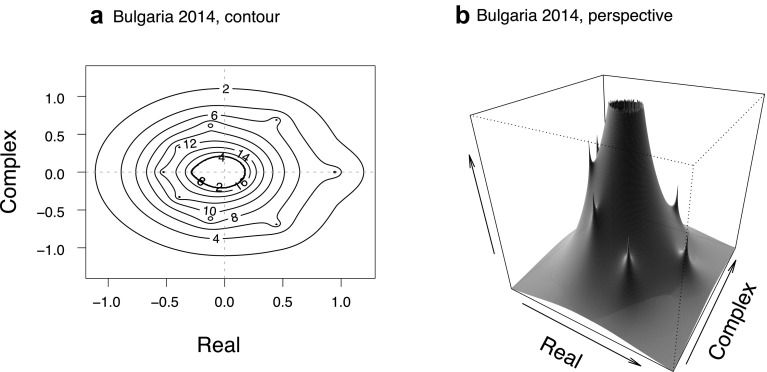
Pseudospectra for Bulgaria in 2014, as a contour plot (**a**) and a perspective plot (**b**). Compare to the spectrum in Fig. 1. Contours correspond to perturbations of the original matrix, with an inverse relationship: small-valued contours correspond to large perturbations, and vice versa. The original, unperturbed, eigenvalues have a ‘height’ of infinity (= 1/0): they are seen as dots in the contour plot and sharp peaks in the perspective plot. Eigenvalues encapsulated by lower-valued contours (e.g., contour 4 around *λ*_1_) would shift only under large perturbations, while those encapsulated by higher eigenvalues (e.g., contour 12 around eigenvalues 6–8) are more easily perturbed. Human PPMs have multiple zero eigenvalues, which explains the ‘volcano’ pattern in the perspective plot, as these eigenvalues are sensitive to even small perturbations

Figure 6 shows two different types of plot for pseudospectra corresponding to the spectrum shown in Fig. 1 (for Bulgaria in 2014). Pseudospectra ‘look beyond’ eigenvalues to express how they change under perturbation (Trefethen 1992; Trefethen and Embree 2005). Here it can be helpful to bear in mind that errors in parameter estimation mean that the ‘true’ model may actually lie within the pseudospectral set of slightly perturbed matrices. Pseudospectra can capture transient dynamics more holistically than eigenvalues—“although pseudospectra rarely give an exact answer, they detect and quantify transients that eigenvalues miss” (Trefethen and Embree 2005, p. 135). Another reason we restricted analyses to the Frobenius norm is that it defines a special case where pseudospectra exactly determine matrix norm behaviour (Greenbaum and Trefethen 1993). Inferences about non-normality can be made by studying eigenvalue encapsulation by the pseudospectra contours: the lower the value of contours encapsulating the eigenvalues, the less stable the matrix and the greater its proneness to transient behaviour.

### Concluding remarks

Regardless of the precise way in which PPM non-normality is incorporated into future study, the insight the metrics offer into transient dynamics renders them an instructive addition to the demographer’s toolbox. At the very least, increasing non-normality, with concomitant transient impacts, necessitates a shift away from the prevailing overreliance on asymptotic growth rate and the damping ratio—which we have shown is too closely related to *λ*_1_ (Fig. 3) and too far removed from transient indices (Fig. 5) to be an optimal transient metric. Implications of non-normality are not restricted to short-term dynamics; even longer-term projections, such as the 2100 population size, should consider transients due to their enduring inertial effect that echoes across generations into the future (Koons et al. 2007). Matrix non-normality measures the extent of the amplificatory impacts of the PPM on the population projection, moving beyond the current focus of demographic projections incorporating transient dynamics solely due to their conditional definition from a specified initial population (Yearsley 2004; Caswell and Sánchez Gassen 2015).

Increasing non-normality suggests intensifying transient effects, with repercussions for European human populations and beyond. Further development of the non-normality metrics applied here (especially our favoured Henrici), along with exploration of pseudospectra, would facilitate improved evidence-based understanding of how the inevitable disturbances that divert population trajectories alter our demographic destinies. Such insight would benefit varied fields, from evolutionary demography (e.g., Metcalf and Pavard 2007), through development studies (e.g., Osotimehin 2011), to population health (e.g., Harper 2010; Kassebaum et al. 2016). Population ecology has long benefitted from an acute awareness of mathematical knowledge. We encourage judicious use of interdisciplinary approaches to help population projection models remain relevant in a continuously changing world.

## Footnotes

1 For readers unfamiliar with eigenvalues and eigenvectors, we recommend the following webpage: http://setosa.io/ev/eigenvectors-and-eigenvalues/.
